# Exome-Wide Association Study Reveals Host Genetic Variants Likely Associated with the Severity of COVID-19 in Patients of European Ancestry

**DOI:** 10.3390/life12091300

**Published:** 2022-08-24

**Authors:** Priyanka Upadhyai, Pooja U. Shenoy, Bhavya Banjan, Mohammed F. Albeshr, Shahid Mahboob, Irfan Manzoor, Ranajit Das

**Affiliations:** 1 Department of Medical Genetics, Kasturba Medical College, Manipal, Manipal Academy of Higher Education, Manipal 576104, India; 2 Yenepoya Research Centre, Yenepoya (Deemed to be University), Mangalore 575018, India; 3 Manipal School of Life Sciences, Manipal Academy of Higher Education, Manipal 576104, India; 4 Department of Zoology, College of Science, King Saud University, Riyadh 11451, Saudi Arabia; 5 Department of Biology, The College of Arts and Sciences, Indiana University, Bloomington, IN 47405, USA

**Keywords:** COVID-19 host genetics, genetic variation in COVID-19 patients, exome-wide association study for COVID-19 patients, common genetic variants

## Abstract

Host genetic variability plays a pivotal role in modulating COVID-19 clinical outcomes. Despite the functional relevance of protein-coding regions, rare variants located here are less likely to completely explain the considerable numbers of acutely affected COVID-19 patients worldwide. Using an exome-wide association approach, with individuals of European descent, we sought to identify common coding variants linked with variation in COVID-19 severity. Herein, *cohort 1* compared non-hospitalized (controls) and hospitalized (cases) individuals, and in *cohort 2*, hospitalized subjects requiring respiratory support (cases) were compared to those not requiring it (controls). 229 and 111 variants differed significantly between cases and controls in *cohorts 1* and *2*, respectively. This included *FBXO34*, *CNTN2,* and *TMCC2* previously linked with COVID-19 severity using association studies. Overall, we report SNPs in 26 known and 12 novel candidate genes with strong molecular evidence implicating them in the pathophysiology of life-threatening COVID-19 and post-recovery sequelae. Of these few notable known genes include, *HLA-DQB1, AHSG, ALOX5AP, MUC5AC, SMPD1*, *SPG7, SPEG,* *GAS6,* and *SERPINA12*. These results enhance our understanding of the pathomechanisms underlying the COVID-19 clinical spectrum and may be exploited to prioritize biomarkers for predicting disease severity, as well as to improve treatment strategies in individuals of European ancestry.

## 1. Introduction

More than two years after its first outbreak, the coronavirus disease (COVID-19) caused by severe acute respiratory syndrome coronavirus 2 (SARS-CoV-2) remains a prominent healthcare challenge and disruptor of social and economic activities worldwide. It shows a complex array of manifestations ranging from the complete absence of symptoms to severe clinical outcomes, such as acute respiratory distress syndrome (ARDS) [1]. It is a multi-systemic disease often marked by cardiovascular, respiratory, neurological, gastrointestinal, renal, immunological, and hematological derangements [2,3,4,5,6,7]. The pathophysiological course of COVID-19 is modulated by the host immune response and progression to severe disease or death occurs due to an uncontrolled cytokine storm and hyperinflammation of the immune system [8]. The severity of clinical outcomes in COVID-19 patients are likely modified by multiple factors ranging from advanced age, male gender, and preexisting comorbidities, such as hypertension and obesity [9,10,11,12]. Moreover, ethnicity-specific differences are also noted in the clinical phenotype of SARS-CoV-2 infection [13,14]. However, these, as well as the discrepancies in socioeconomic attributes or access to healthcare and vaccination do not completely explain the striking individual and population specific disparities observed in the acuteness of COVID-19 clinical presentation worldwide [15,16,17]. For example, many SARS-CoV-2 infected individuals are known to remain asymptomatic despite significantly higher viral loads than their symptomatic counterparts [18]. Furthermore, middle-aged individuals (40–59 years) are known to develop severe disease even without the presence of underlying comorbidities [19]. The spectrum of COVID-19 clinical manifestation is multifactorial and plausibly explained, at least in part, by the variation in host genetic attributes.

This has been interrogated in a series of genome-wide association studies (GWASs) comparing COVID-19 patients and population controls. Ellinghaus et al., reported a significant association of variants in six candidate genes at chromosome 3p21.31, including *Solute Carrier Family 6 Member 20* (*SLC6A20), Leucine Zipper Transcription Factor Like 1 (LZTFL1), C-C Motif Chemokine Receptor 9 (CCR9), C-X-C Motif Chemokine Receptor 6 (CXCR6), FYVE and Coiled-coil Domain Autophagy Adaptor 1 (FYCO1),* and *X-C Motif Chemokine Receptor 1 (XCR1)* and the *ABO* blood group locus at 9q34.2, with respiratory failure in COVID-19 patients from Italy and Spain [20]. These results were corroborated by an independent study in a cohort of SARS-CoV-2 infected individuals comprising of European, African–American and Latino ancestries [21]. Genetics of Mortality in Critical Care (GenOMICC) reported a robust association of single nucleotide polymorphisms (SNPs) within or proximal to genes, such as the *2′-5′ Oligoadenylate Synthetase* (*OAS)* cluster and *Interferon Alpha and Beta Receptor Subunit 2* (*IFNAR2)* on chromosomes 12 and 21, respectively, that are involved in the antiviral type 1 interferon (IFN) response [22,23], *Tyrosine Kinase 2* (*TYK2*), required for IFN, interleukin 12 (IL-12), IL-23 and *T*-helper 1/*T*-helper 17 cell-dependent immunity [24], and *Dipeptidyl Peptidase 9* (*DPP9*), involved in antigen presentation [25], on chromosome 19 with severe outcomes in COVID-19 patients [26]. The COVID-19 Host Genetics Initiative (HG1) report concurred with the association of variants in *ABO*, *SLC6A20, TYK2, DPP9, IFNAR2*, and additionally reported a variant in *Protein Phosphatase 1 Regulatory Subunit 15A* (*PPP1R15A)* in influencing COVID-19 severity [27].

Since uninfected individuals or population controls may develop morbid COVID-19 subsequently upon contracting SARS-CoV-2 infection, we used asymptomatic COVID-19 patients as controls in a GWAS using a COVID-19 dataset generated by AncestryDNA [28], to uncover the genetic variants governing susceptibility to severe COVID-19 in individuals of European ancestry [29]. This study identified 621 SNPs that differed significantly between asymptomatic and acutely afflicted COVID-19 patients, and were associated with IFN and IL signaling, as well as obesity and cholesterol metabolism that are well-known COVID-19 comorbidities. In addition, it highlighted the putative ancestral genomic differences between patients with exacerbated COVID-19 phenotype versus those that were asymptomatic; the latter contained higher genomic fractions of Ancestral North Eurasian (ANE) and Eastern Hunter-Gatherer (EHG), and lower Western Hunter–Gatherer (WHG) fractions. A similar approach using asymptomatic individuals as controls was used in a GWAS on the Chinese population that revealed significant associations at the chromosome loci, 11q23.3 and 11q14.2 with severe COVID-19 and noted significant expression quantitative trail locus (eQTL) associations for RNA Exonuclease 2 (*REXO2), C11orf71*, *Nicotinamide N-Methyltransferase* (*NNMT1),* and *Cell Adhesion Molecule 1 Precursor* (*CADM1)* at 11q23.3 and *Cathepsin C* (*CTSC)* at 11q14.2 [30]. Another trans-ancestry GWAS of Europeans, South Asian, and East Asians from UAE used non-hospitalized COVID-19 patients as controls comparing them to those that were hospitalized [31]. This revealed prominent associations at *Von Willebrand Factor A Domain containing 8* (*VWA8;* 13p11.2), *Phosphodiesterase 8B* (*PDE8B*; 5q13.3), *CTSC* (11q14.2), *Thrombospondin Type 1 Domain containing 7B (THSD7B*; 2q22.1), *Serine–Threonine kinase 39* (*STK39*; 2q24.3), *F-box Protein 34* (*FBXO34*; 14q22.3), *Ribosomal Protein L6 Pseudogene 27 (RPL6P27*; 18p11.31) and *Methyltransferase 21C, AARS1 Lysine* (*METTL21C*; 13q33.3) that are expressed in the lung and associated with lung surface tension, airway obstruction, emphysema, T-cell mediated inflammation, and inflammatory cytokines [31].

Genetic determinants are known to modify the susceptibility to immune-mediated disorders and severe viral infections, such as from respiratory viruses, eg. Influenza virus A and SARS-CoV-1 [32,33,34,35]. As in case of other complex diseases, in addition to a large number of variants in regulatory elements and non-coding regions of the genome [36,37], variations in the coding regions may be involved in controlling the spectrum and severity of COVID-19 [38]. Already rare pathogenic single nucleotide variants in genes governing host immunity, such as *Toll Like Receptor 7* (*TLR7), Toll Like Receptor Adaptor Molecule 1 (TICAM1), Interferon Regulatory Factor 3 (IRF3),* and *Interferon Alpha and Beta Receptor Subunit 1* (*IFNAR1)* are reported in severe cases of COVID-19, including in young patients without pre-existing comorbidities [39,40]. Nevertheless, the monogenic inheritance of rare variants is unlikely to completely account for the large numbers of patients with life-threatening complications in COVID-19 worldwide.

Accordingly, we conducted a case–control based association study to query an exome dataset of 1,432,135 SNPs in 2692 COVID-19 patients of European ancestry from the GEN-COVID consortium, University of Sienna, Italy. We sought to identify common variations in the coding regions of the genome that may be associated with variable prognosis in SARS-CoV-2 infected patients. To this end we employed two strategies, first in *cohort 1* we compared non-hospitalized patients (controls; *N* = 493) to those that were hospitalized (cases; *N* = 2199) and in *cohort 2* we evaluated hospitalized COVID-19 patients on respiratory support (cases; *N* = 1877) to those not requiring the same (controls; *N* = 815).

## 2. Materials and Methods

### 2.1. Dataset

A novel whole-exome dataset of COVID-19 patients was obtained from the GEN-COVID consortium, University of Siena, Italy (Sienna_COVID). It comprised of the data from 2960 COVID-19 patients and assessed 1,432,135 SNPs. The patients were graded on a scale of 0–5 based on their hospitalization status and the requirement of respiratory support (Table 1).

The Sienna_COVID dataset was first merged with the genomic data of individuals from the 1000 Genomes Project (https://www.internationalgenome.org/data/, accessed on 22 April 2017). The merged dataset (Sienna_1K) comprised of 5464 individuals assessing 229,021 SNPs that were common between the two datasets. VCFtools v0.1.16 [41], and PLINK v1.90 [42], were used for all file conversions and manipulations.

**Figure 1:**
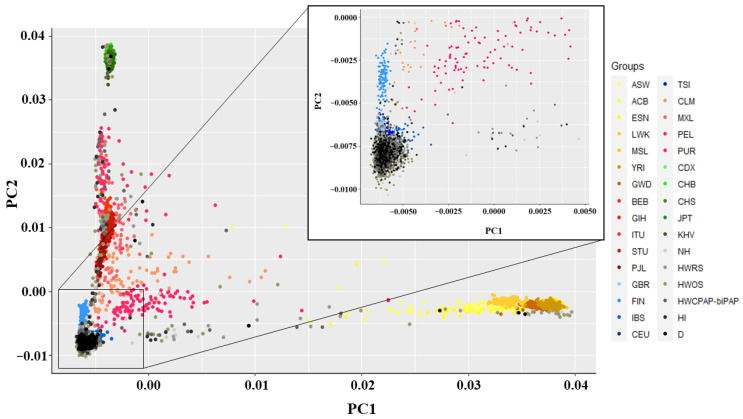
Principal Component Analysis (PCA) of COVID-19 patient genomes. PCA plot showing genetic differentiation among COVID-19 patient genomes. COVID-19 patients (NH, HWRS, HWOS, HWCB, HI, and D) were designated in various shades of grey and dead individuals were designated with black. African, South Asian, European, Latin American, and East Asian populations were designated with different shades of yellow, red, blue, pink, and green, respectively. We selected COVID-19 patients that cluster with European genomes (PC1 ranging from −0.0050 to 0.0050 and PC2 ranging from −0.0100 to 0) for downstream analysis. PCA was performed in PLINK v1.9. The PC1 and PC2 were plotted in RStudio v1.4.1717.

Population structure within the Sienna_1K dataset was identified using Principal Component Analysis (PCA) implemented in PLINK v1.9 using—pca command. The PC1 and PC2 are plotted in RStudio v1.4.1717 (Figure 1). To control for population stratification and avoid genetic structure in the sample, we restricted our analysis to the COVID-19 patients that cluster with individuals of European ancestry from the 1000 Genomes Project [43]. Coordinates for the patients under analysis were delineated based on the clusters formed by GBR, FIN, IBS, CEU, and TSI genomes. 2692 COVID-19 patient exomes within the European cluster were selected (PC1 ranging from −0.0050 to 0.0050 and PC2 ranging from −0.0100 to 0) for downstream analysis (Figure 1 inset), and those outside this cluster were removed. Congruently, the data for 2692 COVID-19 patients of European descents were extracted from the original dataset (Sienna_COVID) and a new dataset assessing 1,432,135 SNPs was generated (European_Sienna_COVID).

Among the 2692 patients considered, the number of individuals in each of the categories described here (Table 1) are Non-Hospitalized (NH; *N* = 493), Hospitalized Without Respiratory Support (HWRS; *N* = 322), Hospitalized Requiring Oxygen Supplementation (HWOS; *N* = 882), Hospitalized Intubated (HI; *N* = 197), Hospitalized With CPAP-biPAP patients (HWCB; *N* = 611), and Dead (D; *N* = 187).

### 2.2. Exome-Wide Association Studies

To identify genetic variants with significant frequency variation between hospitalized (HWRS + HWOS + HWCB + HI + D) versus non-hospitalized (NH) patients (*cohort 1*), and hospitalized patients on respiratory support (HWOS + HWCB + HI + D) versus those without it (NH + HWRS) (*cohort 2*), multiple regression-based case–control analyses were performed on the European_Sienna_COVID dataset in PLINK v1.9. Age, sex, and the first two principal components (PC1 and PC2) were employed as the covariates in the association analysis. To this end, COVID-19 patients were divided into two age groups: ≤50 years and >50 years. All covariates were included in a logistic regression model using --covar flag alongside the --logistic command. SNP-phenotype association was statistically delineated separately for all 1,432,135 SNPs to obtain Odd’s Ratio and respective *p*-value for each SNP after controlling for the covariates. The *p*-value < 0.001 was considered statistically significant. The −log10 *p*-values of all assessed SNPs for both *cohort 1* and *cohort 2* were plotted as Manhattan plots using ‘qqman’ package in R v3.5.2 [44]. Significant SNPs were annotated using SNPnexus (https://www.snp-nexus.org/v4/, accessed on 15 August 2022) web-based server for GRCh38/hg38 [45].

## 3. Results

### 3.1. Exome-Wide Association Analyses

**Figure 2:**
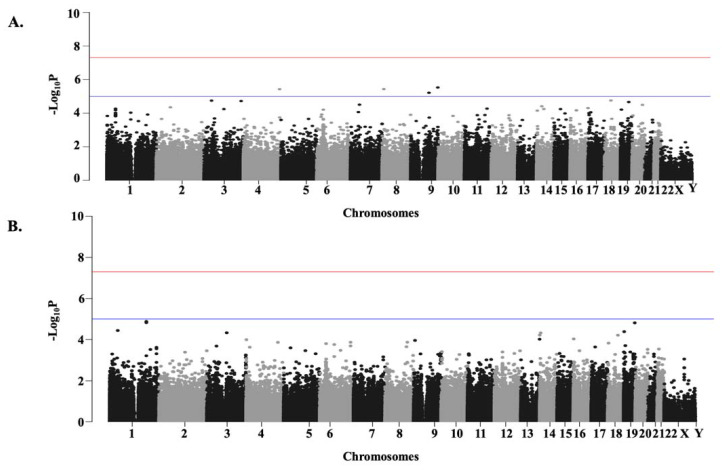
Manhattan Plots summarizing Exome-wide Association Study results. *X*-axis represents chromosomes (chr 1 to chr Y). SNPs present in the chromosomes are designated with dots. Negative log-transformed (−log10) covariate adjusted *p*-values are plotted in the *Y*-axis. The SNPs with *p*-value < 0.00001 are indicated with the blue line, and those with *p*-value < 0.0000001 are indicated with the red line. (**A**). *cohort 1*. Genomes of non-hospitalized COVID-19 patients (*N* = 493) were compared against the hospitalized patients (*N* = 2199). (**B**). *cohort 2*. Genomes of COVID-19 patients with respiratory support (*N* = 1877) were compared against those without respiratory support (*N* = 815).

We assessed the genomes of non-hospitalized COVID-19 patients (*N* = 493; controls) against those hospitalized (*N* = 2199; cases) in *cohort 1*. And compared hospitalized COVID-19 patients on respiratory support (*N* = 1877; cases) with those not requiring it (*N* = 815; controls) in *cohort 2*. Out of 1,432,135 SNPs employed in exome-wide genetic analysis, 229 (*cohort 1*) and 111 (*cohort 2*) SNPs showed significant association with the severity of COVID-19 (*p*-value < 0.001) after controlling for the covariates (Figure 2A and Figure 2B, respectively).

The top 20 highly significant SNPs for *cohorts 1* and *2* are listed in Table 2 and Table 3, respectively with their rsIDs, associated genes, Odd’s Ratio (OR), and *p*-value (*P*).

**Figure 3:**
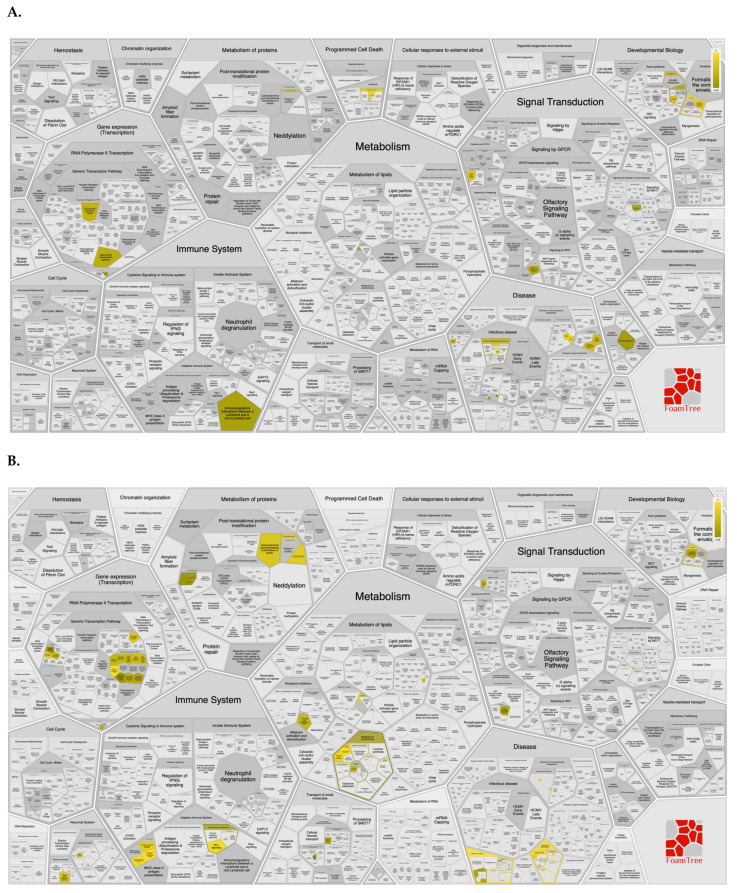
FoamTree representing various Reactome pathways associated with the significant SNPs identified in (**A**). *cohort 1* and (**B**). *cohort 2*. Pathway map was generated using SNPnexus web-based server. Pathways associated with the submitted dataset are highlighted in various shades of yellow. The gray entities represent the pathways that are represented in the query dataset but absent in the submitted dataset.

SNPs that showed highly significant association (*p*-value < 0.001) in *cohort 1* were found to be associated with pathways (https://reactome.org/, accessed on 15 August 2022), such as infectious diseases, NOTCH signaling, immunoregulatory interactions between lymphoid and non-lymphoid cells, and extracellular matrix (ECM) organization (Figure 3A). SNPs with highly significant association (*p*-value < 0.001) in *cohort 2*, were linked with pathways involved in regulation of major histocompatibility complex (MHC) class II signaling, adaptive immune system modulation, carbohydrate metabolism, and RUNX transcription factor mediated regulation (Figure 3B).

Among the SNPs that varied significantly between cases and controls, 11 were found to be common between *cohorts 1* and *2* (Table 4).

Overall, we identified variants in 26 genes with strong links to COVID-19 severity via case–control or cellular studies (Table 5). In addition, we report 12 novel candidates with molecular function and supporting evidence highly suggestive of their contribution to COVID-19 pathology and post recovery sequelae.

### 3.2. Pharmacogenomic Annotation of Significant SNPs

SNPs that showed pronounced variation in our exome-wide association analysis were further annotated for their potential impact on drug responses using pharmacogenomic data obtained and curated from PharmGKB [46]. We found two notable SNPs, one on each cohort. According to PharmGKB, rs2228130 is associated with toxicity of gemcitabine, a chemotherapy drug and rs492602 is associated with alcoholism.

## 4. Discussion

Multiple large-scale case–control GWASs have investigated the genetic basis of the disparity in clinical severity in SARS-CoV-2 infected patients using unaffected individuals, population controls [20,26,27], or asymptomatic and mildly affected individuals as controls [29,30,31]. The exome or protein-coding region is a conserved component of the genome that may harbor potentially damaging variants, which may modulate the clinical phenotype in SARS-CoV-2 infection. Consistent with this, rare pathogenic variants in *TLR7* and eight candidate loci, including, *TLR3*, *IRF3*, and *IRF7* governing type I IFN response have been identified in patients with critical COVID-19 [39,40]. Further a meta-analysis of worldwide exome and genome data implicated rare disease-causing variants in *TLR7* in exacerbating the COVID-19 clinical course [47]. In contrast, largescale exome-wide association studies mostly using population controls did not find rare deleterious exonic variations to be significantly enriched in patients with life-threatening COVID-19 [48,49]. Given that COVID-19 is a genetically complex disorder with polygenic risk inheritance, we surmise that rare pathogenic exonic variants with large effect sizes are unlikely to completely explain the unfavorable disease course noted in large numbers of afflicted patients worldwide. Accordingly, we used an exome-wide association study to identify common genetic variants that are enriched in acutely affected COVID-19 patients of European ancestry. First in *cohort 1*, we compared non-hospitalized patients to those hospitalized and second, in *cohort 2* we evaluated hospitalized COVID-19 patients supported by ventilation to those not needing the same.

Our results included SNPs in the genes *FBXO34*, *Contactin 2* (*CNTN2*), and *Transmembrane And Coiled-Coil Domain Family 2* (*TMCC2*) which have been previously linked with COVID-19 severity [31,50]. *FBXO34* showed significant association with the acuteness of SARS-CoV-2 infection primarily in the European ancestry [31]. While its biological functions are not well understood, F-box proteins at large are a component of the Skip1-Cullin 1-F-box (SCF) E3 ubiquitin ligase complex that participate in proteasome-mediated protein turnover, which are manipulated during infection by many viruses including human immunodeficiency virus (HIV) [51]. Moreover, using GWAS, *FBXO34* has also been linked with plasma protein levels in cardiovascular disease risk among Europeans, as well as with blood cell count [52,53,54,55]. *CNTN2* and *TMCC2* occur in the same genomic region that is significantly associated with risk of poor COVID-19 outcomes in individuals with high European ancestry in Brazil [50].

Specifically, in *cohort 1* we identified SNPs in the following genes, *Killer Cell Immunoglobulin like Receptor, Two Ig Domains and Long Cytoplasmic Tail 1* (*KIR2DL1*) that is an inhibitory receptor upregulated in COVID-19 patients with acute respiratory distress reflecting reduced antiviral activity of natural killer cells in them [56]. *MBL Associated Serine Protease 2* (*MASP2*) that promotes complement cascade activation; variants in *MASP2* resulting in its reduced expression are noted in asymptomatic elderly COVID-19 patients [57]. *Alpha-2 HS glycoprotein* (*AHSG)* that modulates inflammation via attenuating macrophage activation and neutrophil degranulation and is significantly downregulated in severe COVID-19 [58]. The mitochondrial *ATPase family AAA domain containing 3A* (*ATAD3A*) that was upregulated in lymph nodes from COVID-19 autopsy cases [59]; pathogenic variants in *ATAD3A* have also been linked to type I interferonopathy [60]. β*-1,3-N-acetyl-glucosaminyltransferase 8* (*B3GNT8*) that encodes for a glycosyltransferase responsible for anchor point creation in poly-N-acetyl-lactosamine, glycan extensions that could modulate SARS-CoV-2 cellular invasion. Rare variants in *B3GNT8* are linked with milder COVID-19 presentations [61]. *Arachidonate 5-Lipoxygenase Activating protein* (*ALOX5AP*) encodes for 5-LOX activating protein (FLAP), an activating cofactor for the lipid mediator, Arachidonate 5-lipoxygenase (ALOX5) that generates leukotriene B_4_ (LTB_4_) associated with poor respiratory pathologies such as pneumonia, ARDS, and severe lung injury [62,63]. Congruently, increased *ALOX5* activity and *ALOX5AP* expression are observed in bronchoalveolar lavage (BAL) neutrophils in critical COVID-19 patients [64]. Increased expression of *ALOX5AP*, *ALOX5,* and plasma LTB_4_ are also noted in diabetic COVID-19 cases requiring intensive care [65]. *G Protein-Coupled Receptor Class C Group 5 Member C* (*GPRC5C*) whose structural variants have been associated with poor prognosis in COVID-19 [66]. *Mitogen-activated Protein Kinase 7* (*MAP2K7*) encodes for an augmenter of the c-Jun kinase pathway during T-cell activation and is elevated in severe COVID-19 [67,68,69]. *Mucin 5 AC* (*MUC5AC*) that encodes for a gel forming secreted glycoprotein, which is upregulated in the airway mucus of seriously ill SARS-CoV-2 infected patients [70]. *Receptor-interacting kinase 3* (*RIPK3*), a serine–threonine kinase implicated in non-caspase dependent apoptosis termed as necroptosis that leads to ARDS after trauma and sepsis [71]; increased serum levels of RIPK3 are reported in morbid cases of COVID-19 [72]. *Ring Finger Protein 213* (*RNF213*), an E3 ubiquitin ligase and component of the proteasomal degradation machinery that is suppressed in monocytes from severe COVID-19 patients [73]. *Sphingomyelin phosphodiesterase 1* (*SMPD1*) that encodes for a sphinogomyelinase, which catalyzes the synthesis of ceramide, a bioactive lipid mediator involved in response to cellular damage. Upregulation of SMPD1 activity and ceramide have been noted in COVID-19 patients requiring intensive care [74]. *Spastic Paraplegia 7* (*SPG7*) shows increased expression upon SARS-CoV-2 infection [75]. It encodes for a component of the mitochondrial permeability transition pore (mPTP) that is strongly elevated in critical COVID-19 patients and correlated with increased levels of cardiac injury markers in them [76]. Further SARS-CoV-2 proteins localize to the mitochondria, and directly interact with SPG7 to disrupt mitochondrial morphology, energetics, and function in cardiomyocytes [76].

In *cohort 2* our results included SNPs in the following genes, *Kruppel Like Factor 6* (*KLF6*) that encodes for an inflammation regulator with high expression in the macrophages in BAL from grievously ill COVID-19 patients [77]. *Ubiquilin like* (*UBQLNL*) that encodes for a regulator of proteostasis and showed enhanced expression in seriously ill COVID-19 patients [78]. *Fucosyltransferase 4* (*FUT4*), a marker for premature/immature neutrophils that showed elevated expression in severe SARS-CoV-2 infection and is likely associated with poor outcomes in sepsis [79,80]. *Growth-Arrest Specific 6* (*GAS6*) that encodes for a ligand functional in the restorative program initiated to counterbalance pro-inflammatory immune response [81]. GAS6 levels were found to be elevated following SARS-CoV-2 infection and increased with exacerbation of severity [82]. Notably patients with high levels of GAS6 at initial stages were found to have the worst disease prognosis [82]. Interestingly, *GAS6-AS1* encodes for a long non-coding RNA that is downregulated in SARS-CoV-2 infection in vitro and likely interacts with immune modulatory and SARS-CoV-2 interacting proteins such as Adenosine Deaminase RNA Specific (ADAR) and A-Kinase Anchoring Protein 8 Like (AKAP8L) [83]. *Serpin Family A Member 12* (*SERPINA12*) that is involved in the inhibition of kallikrein-dependent inflammation and is downregulated in critical cases of COVID-19 promoting uncontrolled inflammation and worsening of disease outcome [84,85]. *Integrin subunit alpha L* (*ITGAL*), an integrin involved in monocyte migration across endothelium in anti-viral immune response was strongly activated in non-resident macrophages in severe COVID-19 [86]. *Striated Muscle Enriched Protein Kinase* (*SPEG*) that is associated with cardiomyopathy and COVID-19 mortality [87,88]. *Mucin 4* (*MUC4*) that is downregulated in the blood of critically ill COVID-19 patients [89].

The MHC class I and II performs pivotal roles in the host adaptive immunity by modulating the antigen presentation on the cell surface for T-cell recognition [90]. Genetic variation in *human leukocyte antigens* (*HLA*) at the MHC loci modifies the immune response to viral infections, including those caused by SARS-CoV-1 [91], influenza [92], and Middle East respiratory syndrome (MERS) [93]. We identified SNPs in Class II HLA genes, *HLA-DRB1,* and *HLA-DQB1* previously noted to be strongly repressed in a dominant population of dendritic cells [94], in acute cases of COVID-19 [95], in *cohort 1* and *2*, respectively. Reduced allele frequency of *HLA-DRB1* has also been observed in severe COVID-19 [96]. In addition, our results in *cohort 1* included *HLA-DOB,* involved in antigen processing and loading. Its alleles are overrepresented in symptomatic SARS-CoV-2 infected females in a Brazilian cohort [97].

Other findings of interest included variants in novel candidate genes, so far not demonstrated to be directly associated with variability in COVID-19 presentation but with strong accessory evidence for the same. These include *Lysine Acetyltransferase 2B* (*KAT2B*), an epigenetic regulator of TGFβ signaling that is a key pathway in cardiovascular development and disease [98]. Interestingly, SARS-CoV-2 infection is known to trigger aberrant TGFβ signaling that, in turn, mounts a chronic and sustained immune response likely exacerbating disease prognosis [99]. While the molecular mechanisms of KAT2B-dependent regulation of the TGFβ pathway in COVID-19 remain to be understood we surmise host variants in *KAT2B* may modify disease progression and severity following SARS-CoV-2 infection. *One Cut Homeobox 1* (*ONECUT1*) encodes for a transcription factor enriched in the liver and variants in it are associated with different forms of diabetes that is a known risk factor for severe outcomes in COVID-19 [100,101]. *Misshapen (Msn)/NIK related kinase 1* (*MINK1*) encodes for a component of the MAP kinase cascade that is involved in the phosphorylation and priming of the nucleotide-binding domain, leucine-rich-repeat containing family, pyrin-domain containing 3 (NLRP3) inflammasome [102]. Notably NLRP3 activation is central to inflammation and the pathogenesis of ARDS in severe COVID-19 [103]. *Spectrin alpha, Erythrocytic 1* (*SPTA1*) encodes for a structural protein in red blood cells (RBCs). In SARS-CoV-2 infected individuals increased levels and oxidation of SPTA1 peptides are noted reflecting structural aberration of RBCs [104], which may potentially contribute to the severity of hypoxemia, thromboembolism, and coagulation defects noted in the manifestation of COVID-19. *Transporter 2, ATP binding cassette Family B Member* (*TAP2*) encodes for a component of the immunoproteasome that replaces the proteasome in haematopoietic cells, as part of the first line of defense against pathogens following pro-inflammatory cytokine IFN-γ stimulation [105]. Furthermore, high viral load in SARS-CoV-2 infection activates the expression of *TAP2* and other immunoproteasome components in lungs of COVID-19 patients and may result in worsening of disease prognosis [106]. *Unc-93 Homolog B1, TLR Signaling Regulator* (*UNC93B1*) modulates activation of human plasmatoid predendritic cells, which play an important role in SARS-CoV-2 induced immune response [107]. A detrimental clinical course in COVID-19 pathology has been linked with an outburst of pro-inflammatory processes or a ‘cytokine storm’ [108]. Consistent with this we identified variants in *TGF*β *Induced Factor Homeobox 1* (*TGIF1*), related to IFN signaling that is strongly upregulated following SARS-CoV-2 infection [109]. We also found variants in *Colony subunit factor 2 Receptor Subunit* β (*CSF2RB*) that encodes the common subunit of the receptor for IL-3, IL-5, and granulocyte-monocyte stimulating factor (GM-CSF). Increased levels of various cytokines including GM-CSF occur in later stages of SARS-CoV-2 infection producing a self-amplifying cytokine loop that leads to ARDS and mortality [110]. *Hepatocyte Nuclear Factor 4 alpha* (*HNF4A*) that encodes for an intestinal transcriptional regulator which promotes expression of *Angiotensin 1 Converting Enzyme 2* (*ACE2*) and suppresses *Transmembrane Serine Protease 2* (*TMPRSS2*), which are SARS-CoV-2 receptor and involved in viral protein priming, respectively, in the intestine [111]. Gastrointestinal symptoms of varying severity are noted in subsets of COVID-19 patients [112], and may be associated with variation in *HNF4A* in them.

Our analysis in *cohorts 1* and *2* identified SNPs in 17 and 9 genes, respectively, that are already associated with variability in SARS-CoV-2 infection severity (Table 5). We surmise that this is because *cohort 2* interrogates the entire pool of hospitalized patients distinguished only by the requirement of respiratory support, which has been used as a surrogate for more severe disease in the present study and may be regulated by variations at fewer genetic loci. In contrast, *cohort 1* compares individuals who were critically ill necessitating hospital care versus milder, non-hospitalized patients, which are likely distinguished by larger genetic differences modifying the disease severity. Polymorphisms in *AHSG, B3GNT8, DSG2, CFAP57, HLA-DQB1, ONECUT1,* and *SPG7* were among the top 20 SNPs delineated in *cohort 1*. In *cohort 2* variants in *TGIF1* and *GAS6* were among the most prominent 20 SNPs identified. Notable candidate genes common to both cohorts included *ONECUT1* and *HNF4A*.

Further, we also identified SNPs in genes, such as *FK506 binding protein* (*FKBP6*) that is a component of the synaptonemal complex with an indispensable role in meiotic chromosome pairing and fertility in males [113]. *Dipeptidase 3* (*DPEP3*) that encodes a membrane-bound protein in testicular germ cells, which may be important for testicular function and is downregulated in convalescent male COVID-19 patients, likely contributing to compromised fertility in them [114]. *Desmoglein 2* (*DSG2*) that occurs at the intercalated discs coupling adjacent cardiomyocytes and is implicated in arrhythmogenic cardiomyopathy [115]. High levels of circulating anti-DSG2 autoantibodies have been reported in recuperating COVID-19 male subjects, compared to healthy individuals, as well as a subgroup showed elevated levels compared to those in arrhythmogenic right ventricular cardiomyopathy [116]. These data further suggest that host genetic variability not only modifies the course of COVID-19 severity but may modulate features such as cardiovascular disease and male infertility that are also noted in post-COVID-19 sequelae in some patients [117,118].

Finally, we note the absence of information on the SARS-CoV-2 strains for the enrolled patients as a limitation of this approach. Different SARS-CoV-2 genetic strains are associated with distinct clinical outcomes [119]. Since ‘hospitalization’ has been used as a proxy for severe COVID-19 in this study, variance in hospitalization rates due to different COVID-19 strains can potentially obfuscate results obtained here.

Together with existing literature, our results improve the current understanding of genetic factors modulating the spectrum and gravity of the COVID-19 phenotype in individuals of European descent. The novel findings from this work warrant further validatory analysis and studies to facilitate a comprehensive molecular understanding of the COVID-19 pathology. Together the notable candidates from this work may be useful as biomarkers to inform on adverse prognosis and may facilitate the development and deployment of effective therapies, among COVID-19 patients of European ancestry.

## 5. Conclusions

Using exome-wide genetic analysis with data from individuals of European descent we identified common variants in candidate genes that are significantly associated with the severity of COVID-19. Our findings include *FBXO34*, *CNTN2,* and *TMCC2* discovered earlier in other case–control analyses studies. Overall we report SNPs in 26 genes with existing molecular links and 12 novel candidates with biological function or strong supporting evidences suggestive of a high possibility of involvement in modifying the COVID-19 clinical phenotype. These results not only broaden the current knowledge of molecular mechanisms underlying the COVID-19 pathophysiology but may be utilized in delineating a battery of biomarkers predictive of disease outlook in individuals with European ancestry.

## Figures and Tables

**Table 1:** Gradation of COVID-19 patients employed in this study.

Grade	Hospitalization Status
0	Not hospitalized (NH)
1	Hospitalized without respiratory support (HWRS)
2	Hospitalized with O2 supplementation (HWOS)
3	Hospitalized with CPAP-biPAP (HWCB)
4	Hospitalized intubated (HI)
5	Dead (D)

**Table 2:** Top 20 highly significant SNPs identified in *cohort 1*.

SNP	rsID	Gene	OR	*P*
chr9:136451395:G:A	rs45519739	*SEC16A*	0.3082	2.99 × 10^−6^
chr4:182680666:G:C	rs147269509	*TENM3*	0.2923	3.78 × 10^−6^
chr9:91737515:A:G	rs16907720	*ROR2*	0.5227	6.16 × 10^−6^
chr18:31542836:G:A	rs2278792	*DSG2*	1.623	1.78 × 10^−5^
chr3:37481588:G:A	rs17227748	*ITGA9*	1.631	1.80 × 10^−5^
chr3:186620636:A:C	rs1071592	*AHSG*	0.6521	1.91 × 10^−5^
chr19:41426222:G:A	rs137913069	*B3GNT8*	0.2199	2.19 × 10^−5^
chr7:44966456:ACAGCCCT:A	rs563691323	*MYO1G*	0.08036	3.14 × 10^−5^
chr20:52165170:T:C	rs6126487	*ZFP64*	0.4896	3.24 × 10^−5^
chr14:45247163:C:T	rs149551504	*MIS18BP1*	0.1085	3.92 × 10^−5^
chr2:71434819:G:A	rs1398	*ZNF638*	0.1708	4.49 × 10^−5^
chr16:89508537:G:A	rs187330648	*SPG7*	0.3642	5.00 × 10^−5^
chr11:112961669:A:AT	rs782430131	*NCAM1*	0.3525	5.41 × 10^−5^
chr1:43198448:A:G	rs603560	*CFAP57*	0.7111	5.45 × 10^−5^
chr14:55351384:G:A	rs139920556	*FBXO34*	0.03484	5.69 × 10^−5^
chr3:99830513:C:T	rs115071595	*FILIP1L*	0.2011	5.75 × 10^−5^
chr15:52789603:G:C	rs61735385	*ONECUT1*	0.5803	5.77 × 10^−5^
chr19:4497181:C:T	rs146793578	*HDGFL2*	0.4148	6.17 × 10^−5^
chr6:32666522:A:ACC	rs749944694	*HLA-DQB1*	0.599	6.22 × 10^−5^
chr1:43198547:C:T	rs603123	*CFAP57*	0.7139	6.64 × 10^−5^

**Table 3:** Top 20 highly significant SNPs identified in *cohort 2*.

SNP	rsID	Gene	OR	*P*
chr1:184795690:C:T	rs35704242	*NIBAN1*	2.164	1.32 × 10^−5^
chr1:184795547:G:A	rs35545276	*NIBAN1*	2.153	1.50 × 10^−5^
chr19:55602871:G:A	rs310459	*ZNF524*	1.645	1.54 × 10^−5^
chr1:40819499:C:A	rs33932028	*KCNQ4*	1.506	3.62 × 10^−5^
chr19:2279020:A:T	-	*PEAK3*	2.914	4.11 × 10^−5^
chr3:99830513:C:T	rs115071595	*CMSS1/FILIP1L*	0.2032	4.67 × 10^−5^
chr14:23526719:C:T	rs45503996	*ZFHX2*	0.06768	4.72 × 10^−5^
chr14:18967593:G:A	rs199622050	*POTEM*	0.4634	6.04 × 10^−5^
chr18:50819654:G:T	rs3813089	*MRO*	0.7318	6.13 × 10^−5^
chr16:1443487:G:C	rs112232284	*CCDC154*	3.285	9.29 × 10^−5^
chr13:113828592:C:G	rs8191975	*GAS6*	0.6265	9.58 × 10^−5^
chr4:987108:C:A	rs11248061	*SLC26A1/IDUA*	0.7571	1.01 × 10^−4^
chr9:5892525:C:T	rs2233178	*MLANA*	1.725	1.10 × 10^−4^
chr8:112313866:T:C	rs4308763	*CSMD3*	2.306	1.34 × 10^−4^
chr6:155454846:G:T	rs12195525	*NOX3*	1.446	1.35 × 10^−4^
chr4:159355878:G:GCCCCCCC	-	*RAPGEF2*	2.985	1.36 × 10^−4^
chr18:3450457:C:A	rs238132	*TGIF1*	1.44	1.48 × 10^−4^
chr6:32042795:G:T	rs7742632	*TNXB*	0.4841	1.57 × 10^−4^
chr6:73111290:T:A	rs45536838	*KCNQ5*	0.3815	1.74 × 10^−4^
chr19:8094455:G:C	rs34167077	*FBN3*	0.6772	1.96 × 10^−4^

**Table 4:** The SNPs identified in both *cohort 1* and *cohort 2*.

SNP	rsID	Gene	SNP Association	GO Annotation
chr20:62273292:C:T	rs2236526	*OSBPL2*	-	Cholesterol binding
chr16:1443487:G:C	rs112232284	*CCDC154*	-	Bone mineralization/Odontogenesis
chr14:104587865:G:A	rs144894622	*C14orf180*	-	Enables protein binding
chr19:1000718:C:A	rs139242998	*GRIN3B*	-	Calcium channel activity and ionotropic glutamate receptor activity
chr15:52789603:G:C	rs61735385	*ONECUT1*	-	DNA-binding transcription factor activity and RNA polymerase II-specific
chr8:107996952:G:A	rs716149	*RSPO2*	-	Signaling receptor binding and G protein-coupled receptor binding
chr8:112313866:T:C	rs4308763	*CSMD3*	-	ECM organization
chr4:17693320:A:G	rs3733579	*FAM184B*	-	-
chr20:44413724:C:T	rs1800961	*HNF4A*	-	DNA-binding transcription factor activity and sequence-specific DNA binding
chr6:73111290:T:A	rs45536838	*KCNQ5*	HDL cholesterol levels	Ion channel activity and delayed rectifier potassium channel activity
chr3:99830513:C:T	rs115071595	*CMSS1/FILIP1L*	-	Nucleic acid binding and RNA binding

**Table 5:** Genes identified in *cohorts 1* and *2* in this study that belong to various categories of cellular function and have been previously linked to the variation in severity of COVID-19. PubMed IDs (PMIDs) of these articles are also mentioned.

Molecular Pathway/Related Organelle	Genes	*Cohort 1*	*Cohort 2*	PMIDs
**Class II HLA**	*HLA-DQB1*	Yes	-	33972731
	*HLA-DRB1*	-	Yes	33972731
	*HLA-DOB*	Yes	-	34650566
**Immunoglobulins**	*KIR2DL1*	Yes	-	32612152
**Complement cascade**	*MASP2*	Yes	-	35511137
**Inflammation modulatory**	*AHSG*	Yes	-	34139154
	*KLF6*	-	Yes	33138195
	*FUT4*	-	Yes	33441124, 32810438
	*GAS6*	-	Yes	33810394
**Lipid mediator pathway**	*ALOX5AP*	Yes	-	34366882
	*SMPD1*	Yes	-	34987511
**Mitochondria**	*ATAD3A*	Yes	-	35233572
	*SPG7*	Yes		35005527
**Endoplasmic reticulum**	*TMCC2*	-	Yes	35368071
**Cell surface glycans**	*B3GNT8*	Yes	-	35737812
**Extracellular matrix and cell adhesion**	*CNTN2*	Yes	-	35368071
	*MUC5AC*	Yes	-	32776556
	*MUC4*	-	Yes	34448730
	*ITGAL*	-	Yes	32591762
	*SPEG*	-	Yes	33536081
**Cell signaling**	*GPRC5C*	Yes	-	35036860
	*MAP2K7*	Yes	-	32307550
	*RIPK3*	Yes	-	32753065
**Ubiquitin-Proteasome system**	*FBXO34*	Yes	-	34775353
	*RNF213*	Yes	-	34721400
	*UBQLNL*	-	Yes	34335605

## Data Availability

The data employed in this study can be obtained upon request from the GEN-COVID consortium, University of Siena, Italy.
